# The Role of Activity-Dependent DNA Demethylation in the Adult Brain and in Neurological Disorders

**DOI:** 10.3389/fnmol.2018.00169

**Published:** 2018-05-23

**Authors:** Gonca Bayraktar, Michael R. Kreutz

**Affiliations:** ^1^RG Neuroplasticity, Leibniz Institute for Neurobiology Magdeburg, Germany; ^2^Leibniz Group ‘Dendritic Organelles and Synaptic Function’, Center for Molecular Neurobiology (ZMNH), University Medical Center Hamburg-Eppendorf Hamburg, Germany

**Keywords:** DNA Methylation, GADD45B, gene expression, synaptic plasticity, TET enzymes, base excision repair (BER), neural disorders, neurons

## Abstract

Over the last decade, an increasing number of reports underscored the importance of epigenetic regulations in brain plasticity. Epigenetic elements such as readers, writers and erasers recognize, establish, and remove the epigenetic tags in nucleosomes, respectively. One such regulation concerns DNA-methylation and demethylation, which are highly dynamic and activity-dependent processes even in the adult neurons. It is nowadays widely believed that external stimuli control the methylation marks on the DNA and that such processes serve transcriptional regulation in neurons. In this mini-review, we cover the current knowledge on the regulatory mechanisms controlling in particular DNA demethylation as well as the possible functional consequences in health and disease.

## Introduction

Among several other epigenetic tags, methyl tags on the DNA were generally considered as repressive marks. However, an increasing number of studies showed that the DNA methylation at intergenic regions as well as gene regulatory regions might enhance gene expression (Bayraktar and Kreutz, 2017). How, the key enzymes in DNA methylation, DNA methyltransferases (DNMTs), are differentially regulated and perform the DNA methylation are well characterized (Goll and Bestor, 2005; Bayraktar and Kreutz, 2017). However, the removal of methyl tags from the DNA has been more perplexing. The reversal of DNA methylation can take place passively by diluting the DNA methylation of both copies of the genome following multiple rounds of cell division in the absence of maintenance of DNA methylation (Inoue and Zhang, 2011). In postmitotic neurons, other mechanisms must be in place. Current opinion disfavors the direct removal of the covalent bond between the methyl groups and cytosines (Ooi and Bestor, 2008). A unifying mechanistic process on how active DNA demethylation is still lacking. We, therefore, discuss how active DNA demethylation is achieved by the interplay of DNA oxidative reactions and repair mechanisms.

## Mechanisms of Active DNA Demethylation

Several proteins have been identified that are part of neuronal demethylation machinery. These include Growth Arrest and DNA Damage-inducible (GADD) 45 proteins (GADD45A and GADD45B) that in neurons take part in active DNA demethylation processes (Barreto et al., 2007; Ma et al., 2009). Termed initially as the MyD118 (myeloid differentiation), *Gadd45b* was identified as an immediate gene whose expression was induced following the induction of long-term potentiation (LTP) *in vivo* (Hevroni et al., 1998). GADD45B mediated activity-dependent demethylation was first shown for the promoter of fibroblast growth factor 1, isoform B (Fgf1B) and Brain-derived neurotrophic factor (Bdnf) 9. It is nowadays widely believed that GADD45B contributes to demethylation in conjunction with other modifiers which will be discussed below.

**Figure 1:**
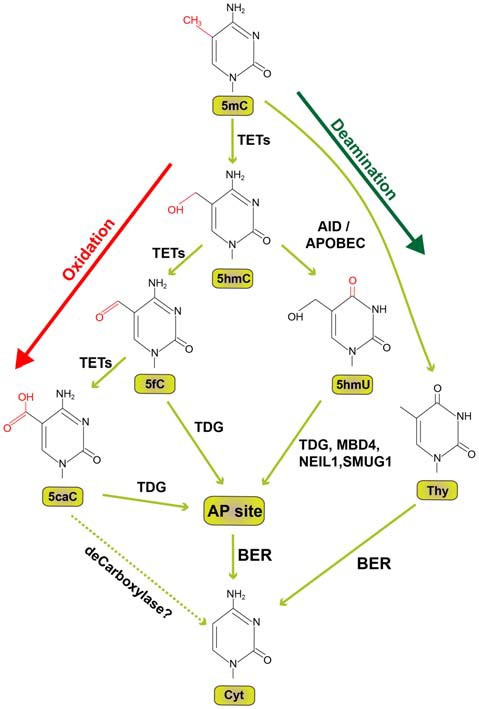
Pathways of active DNA demethylation. Since the formerly hypothesized demethylase to directly convert 5-methylcytosine (5mC) to cytosine (Cyt) has not been identified, we depict here the current view how active DNA demethylation might take place. 5mC is oxidized by ten-eleven translocation (TET) family of dioxygenases to generate 5-hydroxymethylcytosine (5hmC). In successive steps TET enzymes further hydroxylate 5hmC to generate 5-formylcytosine (5fC) and 5-carboxylcytosine (5caC). Thymine DNA glycosylase (TDG) recognizes intermediate DNA forms 5fC and 5caC and excises the glycosidic bond resulting in an apyrimidinic (AP) site. In an alternative deamination pathway 5hmC can be deaminated by activity-induced cytidine deaminase/apolipoprotein B mRNA editing complex (AID/APOBEC) deaminases to form 5-hydroxymethyluracil (5hmU) or 5mC can be converted to Thymine (Thy). 5hmU can be cleaved by TDG, single-strand-selective monofunctional uracil-DNA glycosylase 1 (SMUG1), Nei-Like DNA Glycosylase 1 (NEIL1), or methyl-CpG binding protein 4 (MBD4). AP sites and T:G mismatches can be efficiently repaired by Base Excision Repair (BER) enzymes. Dotted lines indicate a proposed but not experimentally proven path.

5-hydroxymethyl cytosine (5hmC) was first described in the 1972 (Penn et al., 1972) and more than three decades later enzymatic activity of Ten-eleven translocation (TET) proteins was discovered to biochemically convert 5mC to 5hmC (Tahiliani et al., 2009; Ito et al., 2010). The characterization of TET enzymes (Tahiliani et al., 2009; Ito et al., 2010) and 5hmC in the brain (Kriaucionis and Heintz, 2009) also advanced our understanding of active DNA demethylation in neurons. In successive oxidation steps 5mC is initially converted to 5hmC which is followed by the conversion to 5-formylcytosine (5fC) and subsequently 5-carboxylcytosine (5cC). Each of these steps requires one of the three TET enzymes (Ito et al., 2011; Figure 1). 5fC and 5caC can be recognized and excised by Thymine DNA Glycosylase (TDG) generating an apyrimidinic (AP) site (He et al., 2011; Maiti and Drohat, 2011). The AP site is then corrected by specific base-excision repair mechanism (BER) with the replacement of cytosine in mammals (Zhu, 2009). TDG depletion in embryonic stem cells causes enhanced levels of 5fC and 5caC at proximal and distal gene regulatory elements (Raiber et al., 2012; Shen et al., 2013). TDG knockout or catalytical inactivation leads to embryonic lethality in mice and hypermethylated CpG islands (Cortellino et al., 2011). Along these lines, the perturbation of BER enzymes by genetic and pharmacological inhibition results in the partial block of global DNA demethylation in mouse germ line (Hajkova et al., 2010). Collectively this evidence suggests that BER has an evolutionarily conserved role in active DNA demethylation.

**Figure 2:**
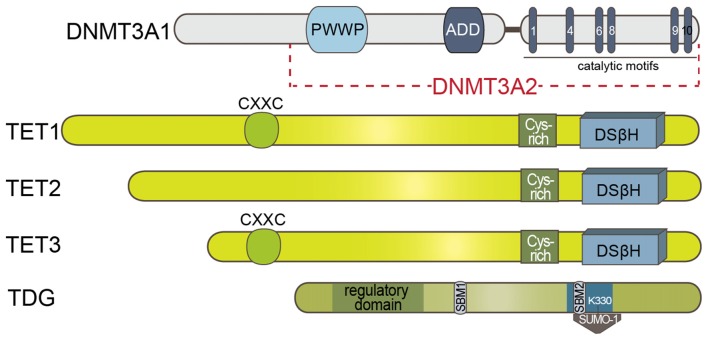
Schematic presentation of the known domain structures of proteins involved in DNA methylation/demethylation. DNMT3A1/2: ADD domain of DNMT3A1/2 is involved in the allosteric control of the enzyme. TET enzymes: DNA binding CXXC motif is present in TET1 and TET3. Double-stranded β-helix (DSβH) is the fold core oxygenase domain is preceded by a cysteine (Cys)-rich domain. Sumo-binding motifs (SBM) and catalytic residues (in blue) of TDG is represented. PWWP: Pro-Trp-Trp-Pro; CXXC: Cys-X-X-Cys motif.

The finding that TDG rapidly processes the oxidation products by TET enzymes corroborated the view of a TET-initiated, TDG-processed and BER-terminated active DNA demethylation mechanism. In support of this picture and the surmised recruiting function of GADD45B, Li et al. (2015) reported that GADD45A as well as GADD45B promote demethylation of an *in vitro* methylated promoter through TDG. TDG physically interacts with GADD45 and in the presence of the triple complex, GADD45B, TDG and TET2, a complete demethylation of reporters could be achieved (Li et al., 2015). TDG has several interesting and yet not well-understood features. It interacts with DNMT3A either via the PWWP or the catalytic domain of DNMT3A (see Figure 2). This interaction enhances TDG activity possibly by facilitating the binding of TDG to the mismatch sites while binding to TDG at the same time represses DNMT3A methyltransferase activity (Li et al., 2007). TDG is one of the two enzymes that together with the methyl-CpG binding domain protein 4 (MBD4) is known to initiate this repair mechanism (Hardeland et al., 2001; Krokan et al., 2002). Both TDG and MBD4 possess low levels of 5mC DNA glycosylase activity *in vitro* (Zhu et al., 2000a,b).

Deamination of 5mC and 5hmC by Activity Induced Cytosine Deaminase (AID) or apolipoprotein B mRNA editing complex (APOBEC) is an alternative path to successive oxidation reactions by TET enzymes for the initiation of DNA demethylation (Figure 1). The modified nucleotide can then be replaced by BER. Shortly after the discovery of AID (Muramatsu et al., 1999), a functional role in DNA demethylation by deamination was proposed (Rai et al., 2008; Bhutani et al., 2010). The identification of AID in a ternary complex with GADD45A and TDG also indicates a contribution of AID in the demethylation process (Cortellino et al., 2011). On the contrary, several independent studies revealed that 5mC and 5hmC are poorer substrates for AID as compared to cytosine (Larijani et al., 2005; Nabel et al., 2012; Rangam et al., 2012; Abdouni et al., 2013). The contribution of AID for the deamination pathway to demethylate 5mC in association with BER mechanism is rather elusive since the enzyme cannot efficiently deaminate 5mC (Wijesinghe and Bhagwat, 2012). However, the same study showed efficient 5mC deamination capability of APOBEC3 (Wijesinghe and Bhagwat, 2012). In conclusion, the role of AID in DNA demethylation particularly in the adult brain is still unclear (for review, see Bochtler et al., 2017).

## Regulation of Neuronal Gene Expression by Active DNA Demethylation

In the long-lived nature of postmitotic neurons, genomic stability needs to be maintained for decades while at the same time their remarkable plasticity has to be kept at a poised state ready to respond (Baker-Andresen et al., 2013). How are stability and permissiveness for changes in DNA methylation achieved upon enhanced neuronal activity? It is tempting to speculate that due to its plastic nature the basal epigenomic state of hippocampal neuron determines the permissiveness for an initial wave of transcription of DNA modifiers, including demethylation machinery component expressions, which precedes effector gene expression (Oliveira, 2016). An example for a methylation mark keeping the gene in a silent but in a transcriptionally poised state is the promoter methylation of the *Bdnf* gene that is quite well investigated in the context of synaptic plasticity and learning (Miller and Sweatt, 2007; Lubin et al., 2008). In differentiated neurons, the *Bdnf* promoter is methylated at basal conditions and thereby kept in a repressed state by the occupation of repressor complex involving RE1-Silencing Transcription factor corepressor (CoRest), methyl CpG binding protein 2 (MeCP2), histone deacetylases (HDAC) 1 and 2. The repressor complex dissociates following phosphorylation of MeCP2 and nitrosylation of HDAC2 in response to Ca^+2^ influx (Chen et al., 2003; Nott et al., 2008). Activity-induced deaminase (AID) regulates the induced expression of *Bdnf IV* in a stimulus-dependent manner (Ratnu et al., 2014). However, the yet unclear status of AID in DNA demethylation makes it hard to directly link the effect of AID on Bdnf expression to activity-dependent DNA demethylation.

Unfortunately, conflicting reports have been published on the role of GADD45B in learning and memory processes. Fear conditioning induces *Gadd45b* gene expression (Keeley et al., 2006) and deletion of the gene results in hippocampus-dependent long-term memory deficits including fear conditioning (Leach et al., 2012). However, others found improved long-term memory following Gadd45b knockout mice employing a similar contextual fear-conditioning paradigm (Sultan et al., 2012). Of note, the mice strains used in the latter two studies had a different genetic background (C57BL/6 and B6:129VJ mice) which might account for the discrepant results. Moreover, targeted siRNA delivery to transiently knock down Gadd45b expression in the neonatal rat amygdala results in altered juvenile behavior with consequences for the expression of Bdnf, MeCP2 and Reelin (Kigar et al., 2015). NF-κB, which is known to be important in hippocampus-dependent memory formation (Kaltschmidt and Kaltschmidt, 2015), was proposed to regulate Gadd45b gene expression and thereby DNA demethylation activity (Jarome et al., 2015). Interestingly, overexpression of TET1 leads to enhanced expression of several memory-related genes but surprisingly to impairment of contextual fear memory (Kaas et al., 2013). It is possible that TET proteins might have functions independent of DNA hydroxymethylation. TET3, for instance, was recently shown to have a functional role in scaling-down synaptic strength in hippocampal neurons (Yu et al., 2015).

Interestingly, 5hmC is not only an intermediate DNA demethylation form but also an epigenetic mark on its own, which is enriched within promoters and gene bodies (Kaas et al., 2013). This enrichment correlates with a depletion of 5mC in actively transcribed genes. Moreover, gene body 5mC and gene expression are inversely correlated (Mellen et al., 2012). Recent advances in whole epigenome analysis identified gradually accumulating non-CG methylation (mCH, H = A/C/T) from post-natal week one onwards in the genome peaking in the adult mouse brain (Xie et al., 2012; Lister et al., 2013) and at several hundred genomic positions in the adult human brain (Varley et al., 2013). Genes expressed in the mammalian brain are devoid of intragenic and promoter mCH (Xie et al., 2012) and mCH correlates with decreased gene expression in a highly cell type-specific manner (Mo et al., 2015). mCH accumulation is implicated in X chromosome inactivation and might therefore contribute to gender specific gene expression (Keown et al., 2017). Reconfiguration of the global DNA methylome during development coincides with synaptogenesis, a period in which mCH accumulates in neurons but not in glial cells (Lister et al., 2013). On the other hand, methylated CpG-rich DNA regions are not only found in transcription initiation sites but also in gene bodies and intergenic regions (Jones, 2012). Collectively these studies illustrate that it is important to identify the location and type of DNA methylation to assess its contribution to gene expression.

Another critical issue is cell-type specificity of DNA demethylation. In most studies brain tissue that contains different neuronal and glial cell types was used. The current knowledge on how the DNA demethylation machinery functions in different cell-types and responds to neuronal activity is therefore very limited. DNA methylation patterns vary between neurons and non-neuronal cells. Ventromedial prefrontal cortex neurons have higher global DNA methylation levels compared to non-neuronal cells (Li et al., 2014a). Early life stress (ELS) alters DNA methylation and Bdnf expression in medial prefrontal cortex neurons in a cell-type and sex-specific manner (Blaze and Roth, 2017). The expression of Bdnf IX and Fgf1B genes, which are crucially involved in neurogenesis and plasticity, is also regulated by Gadd45b in an activity-dependent manner in granule cells of the dentate gyrus (Ma et al., 2009). Furthermore, Halder et al. (2016) showed that DNA methylation and changes in histone acetylation occur in parallel following contextual fear conditioning learning and alterations in DNA methylation may also arise in non-neuronal cells potentially supporting an epigenetic code for memory formation. Interestingly, in contrast to hippocampal neurons, TET3 but not TET1 is expressed in cortical neurons in an activity-dependent manner (Li et al., 2014b). Gephyrin stabilizes GABA receptors to postsynaptic membrane and takes part in fear extinction (Chhatwal et al., 2005). Li et al. (2014b) further validated the enhanced expression of TET3 on the gephyrin locus where they showed increased occupancy of TET3 in association with an accumulation of the demethylation intermediate mark 5hmC.

## Role of DNA Demethylation in Neurological Disorders

Given the principal functions of chromatin modifications in regulating gene transcription programs, it’s not surprising that the number of studies, which report the involvement of DNA demethylation machinery in neurological disorders, is steadily increasing. Enhanced GADD45B levels were reported in two different cohorts of major psychotic patients (Gavin et al., 2012). However, reduced occupancy of GADD45B on the *Bdnf IX* promoter was found, which is in line with reduced *Bdnf IX* expression (Gavin et al., 2012). Recently, GADD45B expression was shown to be regulated by transforming growth factor beta (TGFB) signaling and protein levels of GADD45B are reduced in a model of chronic mild stress (Grassi et al., 2017). Moreover, a reduction in expression levels of the immediate early gene *Arc* was also associated with reduced levels of GADD45B and DNA demethylation in this stress model (Grassi et al., 2017). Although its neurobiological underpinnings have not been fully understood, electroconvulsive therapy (ECT) is currently in clinical practice for the treatment of several psychiatric diseases including depression (Singh and Kar, 2017). In an animal model ECT reduces the methylation levels of Bdnf 9 promoter, hence inducing the mRNA expression of the gene, however, in the transgenic mice model in which Gadd45b was knocked out the effect of ECT on the *Bdnf IX* promoter methylation level is abolished and mRNA expression is perturbed (Ma et al., 2009). Prenatally stressed mice exhibit not only similar behavioral traits like psychotic patients but also similar epigenetic signatures (Dong et al., 2015). DNA methyltransferase1 and TET1 enzyme level increase in prenatally stressed mice correlates with enhanced 5mC and 5hmC in the regulatory DNA regions and hence decreased *Bdnf* gene expression (Dong et al., 2015). Samples from patients that suffered from bipolar disorder and schizophrenia show enhanced methylation of associated gene promoters resulting in suppressed expression (Grayson and Guidotti, 2013). This is linked to the enhanced expression of DNMTs (Veldic et al., 2004; Zhubi et al., 2009). However, it’s not clear whether the lack of active DNA demethylation can also be responsible for the disease etiology in some cases. The contribution of methylation and active DNA demethylation in Alzheimer’s disease (AD) remains to be determined. The varying global methylation levels reported in the postmortem brain samples can be region specific (Roubroeks et al., 2017). There are conflicting studies on the increase in 5mC and 5hmC in the hippocampus whereas no changes in the entorhinal cortex in AD as compared to controls were reported (Bradley-Whitman and Lovell, 2013; Lashley et al., 2015). Contradictory evidence on the global decrease in methylation levels in the hippocampus and entorhinal cortex was published by others (Mastroeni et al., 2010; Chouliaras et al., 2013). In a complex disease like AD, the readout from brain samples and genome-wide association studies on various chromatin modifiers is hard to interpret because of the readout’s variability due to the initiation, progression or terminal stage of the disease.

## Concluding Remarks

Based on the initial GADD45B-dependent demethylation hypothesis (Gavin et al., 2012), current data suggest that active demethylation in postmitotic neurons is initiated by TET family enzymes in conjunction with TDG. While GADD45B apparently lacks enzymatic activity, it seems to recruit demethylation machinery components to certain promoters by yet unknown mechanisms. The cascade of events in active DNA demethylation finally requires the contribution of BER mechanism to generate mark-free cytosine. There are several gaps in our understanding of the DNA demethylation pathway in neurons. For instance, how are the DNA demethylation components targeted to specific genomic sites? Finally, it is yet unclear how one can interfere with this machinery to regulate activity-dependent gene expression and whether this machinery has druggable pathways in the context of neurological disorders.

## Author Contributions

GB and MK are invited to contribute to the article collection for the special issue of the “Epigenetic Mechanisms Regulating Neural Plasticity”. GB outlined the mini review and GB and MK wrote the manuscript.

## Conflict of Interest Statement

The authors declare that the research was conducted in the absence of any commercial or financial relationships that could be construed as a potential conflict of interest.
